# Notch3 overexpression enhances progression and chemoresistance of urothelial carcinoma

**DOI:** 10.18632/oncotarget.16156

**Published:** 2017-03-13

**Authors:** Heng Zhang, Limei Liu, Chungang Liu, Jinhong Pan, Gensheng Lu, Zhansong Zhou, Zhiwen Chen, Cheng Qian

**Affiliations:** ^1^ Institute of Pathology and Southwest Cancer Center, Southwest Hospital, Third Military Medical University, Chongqing 400038, China; ^2^ Department of Urology, Southwest Hospital, Third Military Medical University, Chongqing 400038, China

**Keywords:** Notch3, urothelial cancer, cisplatin, histone deacetylase, chemoresistance

## Abstract

Abnormal activation of Notch signaling is involved in the etiology of various diseases, including cancer, but the association between *Notch3* expression in urothelial cancer and clinical outcome remains unclear, and the molecular mechanisms underlying Notch3 signaling activation are not well defined. In this study we examined 59 urothelial cancer patients and found that *Notch3* was more highly expressed in human urothelial cancer tissues than in non-tumorous bladder tissue samples, with *Notch3* overexpression being associated with poor clinical outcome. *Notch3* knockdown resulted in decreased proliferation of urothelial cancer cells *in vitro* and decreased xenograft tumor growth *in vivo*. In addition, *Notch3* knockdown rendered urothelial cancer cells more sensitive to cisplatin. Furthermore, suberoylanilide hydroxamic acid (SAHA, a histone deacetylase [HDAC] inhibitor) induced acetylation of NOTCH3, downregulated *Notch 3*, prevented urothelial cancer cell proliferation, and induced cell cycle arrest. Taken together, these data suggested that *Notch 3* overexpression promotes growth and chemoresistance in urothelial cancer.

## INTRODUCTION

Bladder cancer is the ninth most common malignancy worldwide, with estimated 386,300 new cases and 150,200 deaths from bladder cancer in 2008 [1]. Epidemiological data in China reveal that bladder cancer still has the highest incidence among tumors of the urinary system, with 14.72/100,000 and 5.34/100,000 in males and females, respectively [2]. In the United States, medical expenses for bladder cancer ranks first among all malignancies, with a mean of $202,000 per patient, i.e. 2 folds compared with lung cancer costs [3]. This imposes a heavy economic burden on the society. The biological processes of bladder cancer are very complex, with several features such as heterochrony and heterotopy, high recurrence, multiple tumors, progression to invasive bladder cancer, and metastasis. Therefore, new therapies are urgently needed to improve patient survival. Targeting agents that promote bladder cancer development and progression could constitute a promising option.

Notch signaling is a conserved pathway involved in several human cancers, including urothelial cancer [4–7]. However, its role in tumorigenesis is highly context-dependent, acting by either promoting or suppressing tumors depending on disease setting [8]. Notch pathway activation relies on ligand-induced proteolytic cleavage of the receptor, which results in the release of the intracellular domain of Notch (NICD). NICD then travels to the nucleus and initiates highly diverse transcriptional programs that control various cellular functions.

Notch3 is required for tumor propagation in mouse models of non-small-cell lung cancer and human non-small-cell lung cancer [9]. In addition, *Notch3* is activated by chronic hypoxia, and contributes to the progression of human prostate cancer [10]. Overexpression of *Notch3* is associated with chemoresistance and poor overall survival of human ovarian cancer patients and induced resistance to carboplatin in ovarian cancer cells [11]. Overexpression of *Notch3* also induced epithelial-mesenchymal transition and attenuated carboplatin-induced apoptosis [12]. These findings suggest that *Notch3* activation contributes to tumor progression and drug resistance, but the role of *Notch3* in urothelial cancer remains unclear and needs to be further investigated.

In this study, we found that high expression of *Notch3* was associated with poor patient survival, and may serve as a prognostic marker for urothelial cancer. Decreasing the expression of *Notch3* using histone deacetylase (HDAC) inhibitors is likely to become an effective therapeutic strategy.

## RESULTS

### Notch3 is highly expressed in human urothelial cancer

**Figure 1:**
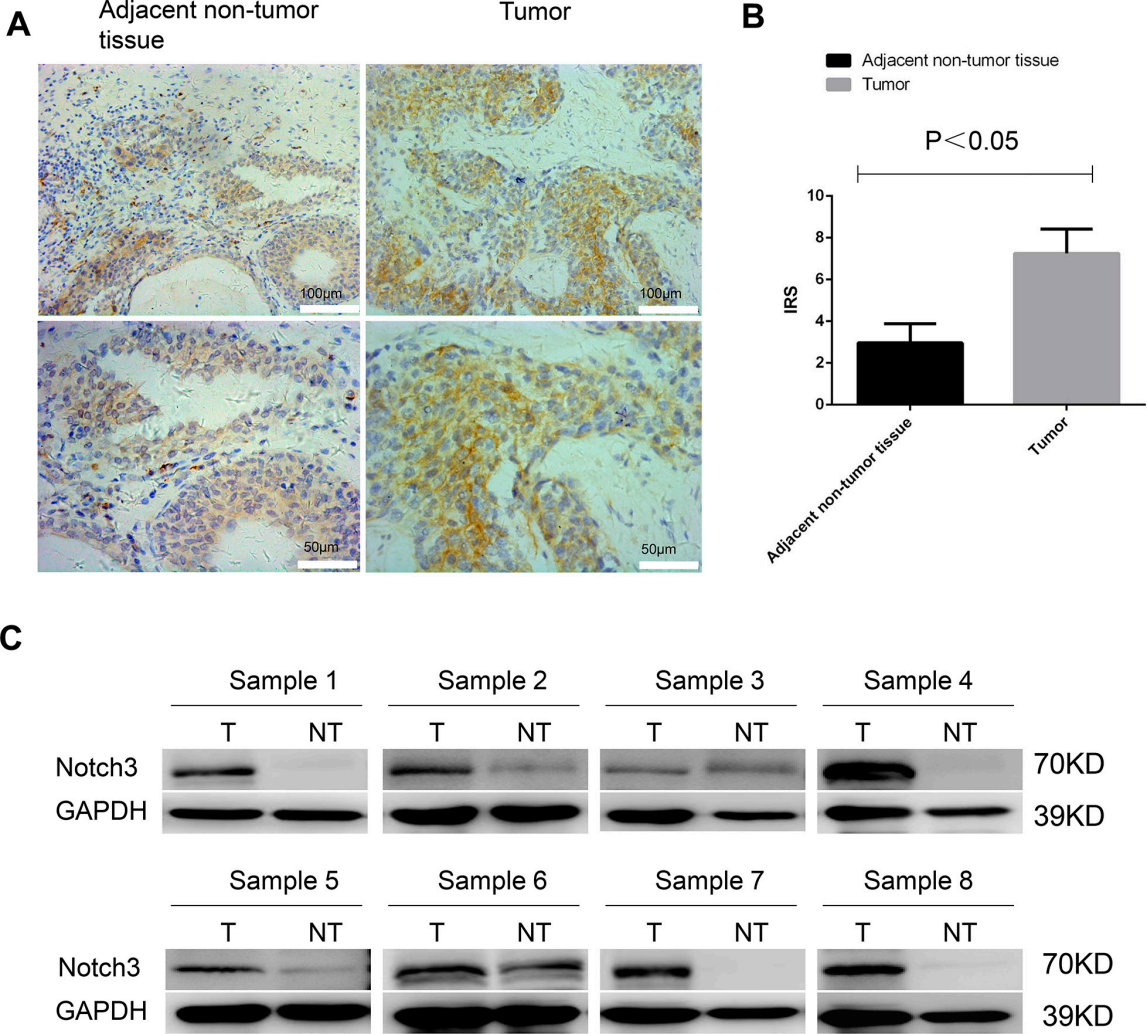
Notch3 is highly expressed in urothelial cancer Notch3 amounts in a tissue array containing 59 paraffin-embedded formalin-fixed urothelial cancer tissue samples and matched non-tumor counterparts (non-tumor tissue from the same patient) were examined by immunohistochemistry. (**A**) Representative photomicrographs of human urothelial cancer specimens. Notch3 was expressed in adjacent non-tumor tissue but that its expression was much higher in tumor tissues. (**B**) Immunoreactive scores (IRS) for Notch3 expression in urothelial cancer tissues (T) compared with non-tumor tissues (NT) from 59 urothelial cancer patients. (**C**) Notch3 protein levels in eight fresh urothelial cancer tumor specimens (T) and matched non-tumor tissue samples (NT) examined by western blot.

To assess *Notch3* expression levels in human urothelial cancer, we first used immunohistochemistry to examine a paraffin-embedded tissue array containing samples from 59 patients with urothelial cancer. The results showed that Notch3 was expressed in adjacent non-tumor tissue but that its expression was much higher in tumor tissues (Figure 1A–1B). Among the 59 urothelial cancer samples examined, two showed lower Notch3 expression than the matched non-tumor tissue (non-tumor tissue from the same patient); four samples showed Notch3 expression level equal to that in matched non-tumor tissues, and 53 samples showed higher levels of Notch3. Representative micrographs and immunoreactive scores (IRS) are displayed in Figure 1A and 1B, respectively. To further assess the levels of Notch3 in urothelial cancer, western blotting analysis was performed on fresh specimens of tumors and matched non-tumor tissues from eight urothelial cancer patients. As shown in Figure 1C, Notch3 protein levels were significantly higher in urothelial cancer tissues compared to non-tumor tissues (*P* < 0.05). These results demonstrated that *Notch3* expression was increased in human urothelial cancer.

### High Notch3 levels are associated with poor prognosis in patients with human urothelial cancer

**Figure 2:**
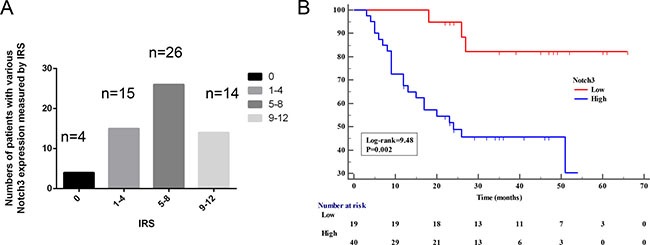
High Notch3 levels are associated with poor prognosis (**A**) Notch3 levels were assessed in 59 paraffin-embedded formalin-fixed urothelial cancer tissues and matched non-tumor tissues (from the same patient) by immunohistochemistry. Bars show the number of patients with no (IRS = 0), low (IRS = 1–4), moderate (IRS = 5–8), and high (IRS = 9–12) expression in urothelial cancer tissues. (**B**) Kaplan-Meier curve for overall survival was compared according to Notch3 levels in human urothelial cancer tissues. Samples were divided as low (IRS < 4) and high (IRS ≥ 4).

Subsequently, we investigated whether Notch3 levels are associated with clinical outcomes in patients with urothelial cancer. Notch3 was detected in 55 out of 59 samples (93.2%) of bladder tumor tissues (Figure 2A). Among the positive samples, 15/55 (27.3%), 26/55 (47.3%), and 14/55 (25.4%) had high (IRS = 9–12), moderate (IRS = 5–8), and low (IRS = 1–4) levels, respectively. Clinical and pathological analyses showed that high Notch3 levels (strong staining) were not significantly associated with tumor size and stage (Table 1), but the Kaplan-Meier analysis indicated that patients with high Notch3 levels had significantly shorter overall survival than those with low levels (Figure 2B, *P* < 0.001).

**Table 1:** Clinical characteristics and Notch3 expression levels in patients with bladder cancer

Variables	*n*	Notch3 expression^a^		*P*-value
		Low (*n* = 19)	High (*n* = 40)	
Age (years)				
< 50	2	1	1	0.544
≥ 50	57	18	39	
Gender				
Female	10	2	8	0.593
Male	49	17	32	
Tumor stage				
Ta	1	1	0	0.584
Tis	2	1	1	
T1	12	5	7	
T2	12	4	8	
T3	20	5	15	
T4	12	3	9	
Tumor size (cm^3^)				
< 5	39	13	26	0.795
≥ 5	20	6	14	
Lymph node positivity				
No	45	15	30	0.996
Yes	14	4	10	
Distant metastasis				
M0	59	19	40	---
Infiltration depth in bladder				
Ta, T_1_, T_is_	15	6	9	
≥ T_2_	44	13	31	
Histological grade				
PUNLMP	8	4	4	0.291
Low	28	10	18	
High	23	5	18	

^a^High = IRS of Notch3 expression > 4 points as assessed by immunohistochemistry; Low = IRS of Notch3 expression < 4 points (See Figure 2).

PUNLMP: papillary urothelial neoplasms of low malignant potential.

### Notch3 knockdown inhibits the proliferation of urothelial cancer cells *in vitro*

**Figure 3:**
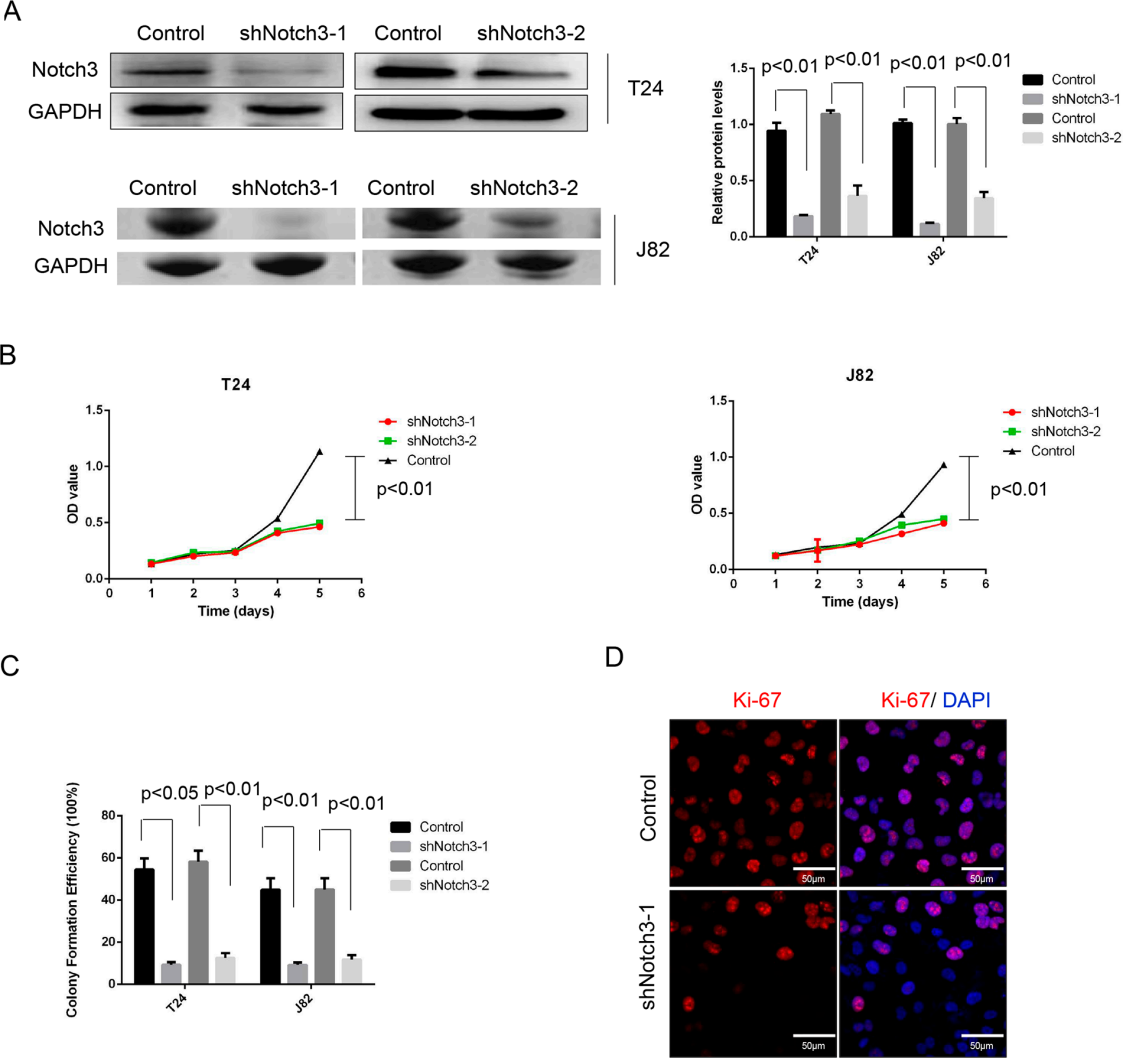
*Notch3* knockdown inhibits cell growth *in vitro* (**A**) Notch3 levels in T24 and J82 cells after treatment with Notch3-shRNAs (shNotch3-1 and shNotch3-2) or scrambled shRNA (control), determined by western blot. (**B**) Cell proliferation of T24 and J82 cells after treatment with Notch3-shRNA or scrambled shRNA was assessed by CCK-8 kit, and mean ± SD from three independent experiments. (**C**) Colony formation efficiency was assessed in T24 and J82 cells after treatment with Notch3-shRNA or scrambled shRNA. Representative photographs from three independent experiments. (**D**) Expression of Ki-67 levels in T24 cells after treatment with Notch3-shRNA or scrambled shRNA, determined by immunofluorescence. Representative photomicrographs from three independent experiments. Scale bar, 50 μm.

To determine whether *Notch3* contributes to urothelial cancer cell proliferation and disease progression, specific shRNAs against *Notch3* (shRNA-Notch3-1 and shRNA-Notch3-2) were used to knockdown *Notch3* in the human urothelial cancer cell lines T24 and J82. *In vitro* proliferation of T24 and J82 cells was assessed after treatment with Notch3-shRNAs or scrambled shRNA (control). We found that *Notch3* expression in cells was dramatically reduced after treatment with both Notch3-shRNAs. However, shRNA-Notch3-1 showed slightly higher efficiency (Figure 3A). Thus, shRNA-Notch3-1 (shRNA-Notch3) was selected for subsequent experiments. Knockdown of *Notch3* in T24 and J82 cells inhibited cell growth (Figure 3B). In addition, colony formation efficiency was decreased compared to the control group (Figure 3C). Furthermore, knockdown of *Notch3* in T24 cells resulted in significantly lower expression of the proliferation marker Ki-67 compared to the control group (Figure 3D). These data indicated that reduced urothelial cancer growth induced by *Notch3* knockdown was due to reduced cell proliferation.

### Notch3 knockdown decreases tumor growth *in vivo*

**Figure 4:**
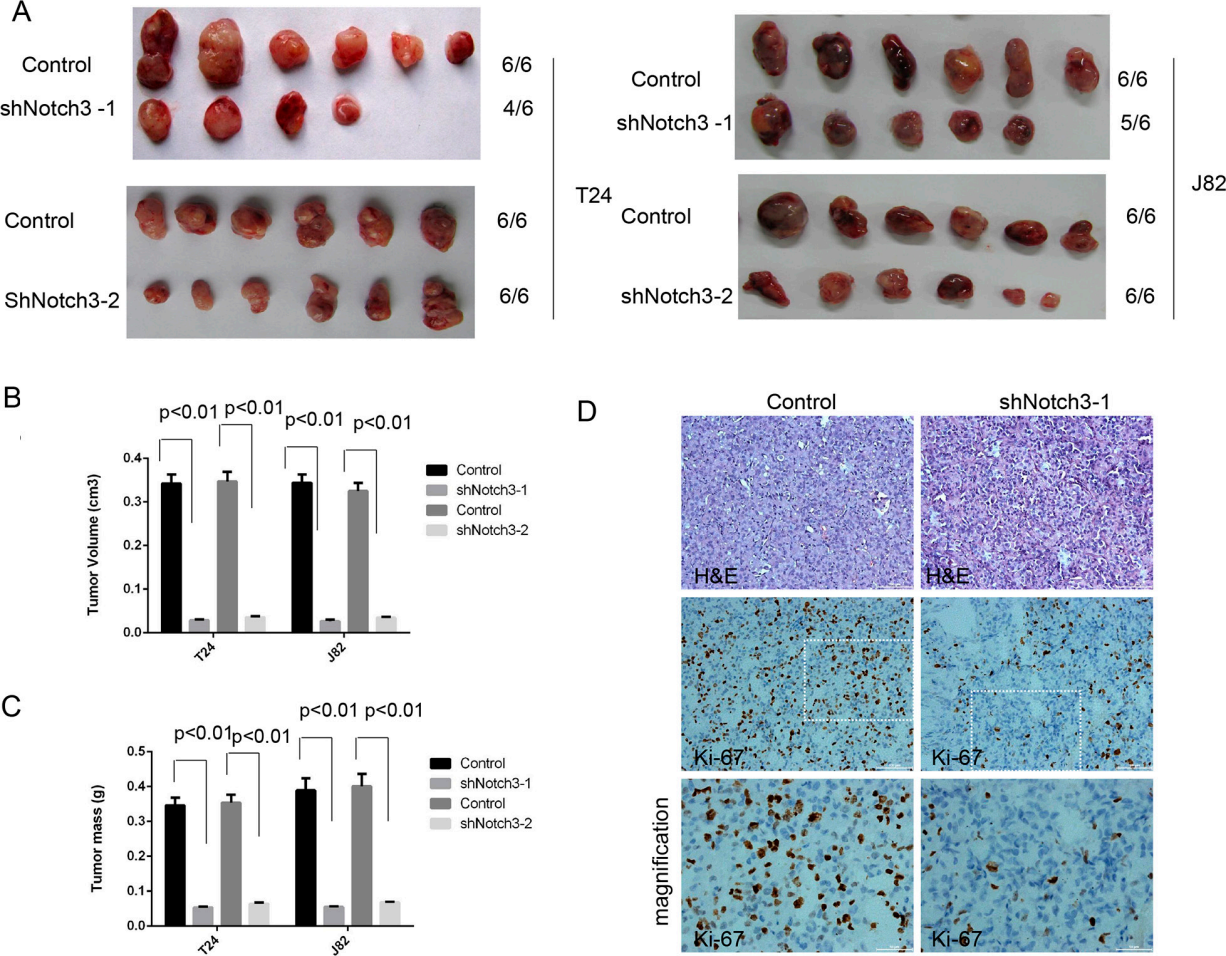
*Notch3* knockdown decreases tumor growth *in vivo* (**A**–**C**) Notch3 promotes tumor growth *in vivo*. A xenograft model was established using T24 and J82 cells with or without *Notch3* knockdown. Four weeks after injection of T24 and J82 cells, mice were sacrificed and tumor volumes and weights were obtained. Data are presented as mean ± SEM (*n* = 6 in each group). (**D**) Ki-67 levels in T24 xenograft tissues after treatment with Notch3-shRNA or scrambled shRNA, determined by immunohistochemistry. Representative photomicrographs are presented. Scale bar, 50 μm.

We next sought to assess whether NOTCH3 could induce similar effects *in vivo*. Therefore, we established a xenograft model by using the bladder cancer cell lines T24 and J82 in NOD/SCID mice. Four weeks after establishment of the xenograft models, tumors generated from T24 and J82 cells with *Notch3* knockdown were smaller than those generated by control T24 and J82 cells (Figure 4A and 4B). In addition, *Notch3* knockdown decreased tumor weight compared to control values (Figure 4C). In agreement, *Notch3* silencing resulted in reduced Ki-67 levels in urothelial cancer cells *in vivo* (Figure 4D). The above findings clearly demonstrated that *Notch3* is necessary for maintaining urothelial cancer cell growth and tumor progression. Therefore, *Notch3* upregulation in urothelial cancer cells could be a critical determinant of overall tumor growth.

### Decreased Notch3 expression sensitizes urothelial cancer cells to cisplatin

**Figure 5:**
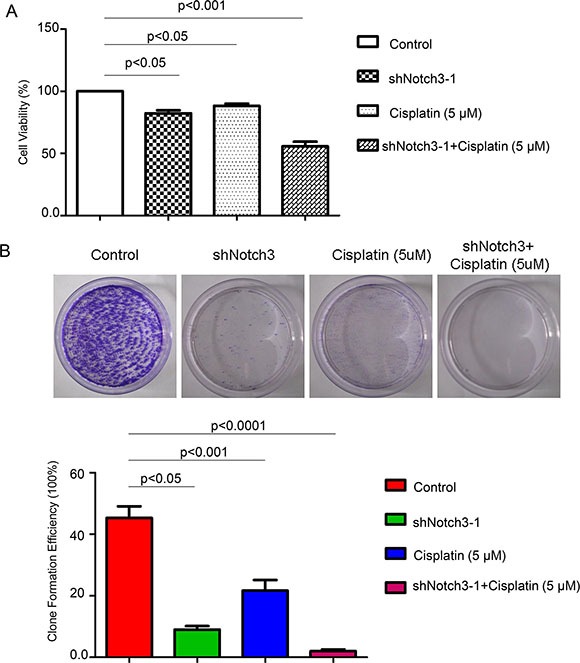
*Notch3* knockdown renders urothelial cancer cells sensitive to a 15-day treatment with cisplatin (**A**) Cell viability of T24 cells under different treatment was detected by CCK-8 assay. (**B**) To assess the formation of cisplatin-resistant colonies, T24 cells were either untreated (control) or treated with Notch3-shRNA alone, cisplatin alone, or cisplatin plus Notch3-shRNA, and cultured for 15 days.

To determine whether *Notch3* knockdown increases the sensitivity of urothelial cancer cells to cisplatin, T24 cells were treated with cisplatin after *Notch3* knockdown. As shown in Figure 5A, both *Notch3* knockdown and treatment with cisplatin (5 μM) decreased cell viability of T24 cells, but the reduction was more pronounced in *Notch3* knockdown cells treated with cisplatin. Colony formation assay showed similar results, with almost complete elimination of cisplatin-resistant colonies observed in *Notch3* knockdown T24 cells (Figure 5B). These findings indicated that inhibition of *Notch3* expression could sensitize urothelial cancer cells to cisplatin.

### Treatment with HDAC inhibitor suppresses Notch3 signaling in urothelial cancer cells

**Figure 6:**
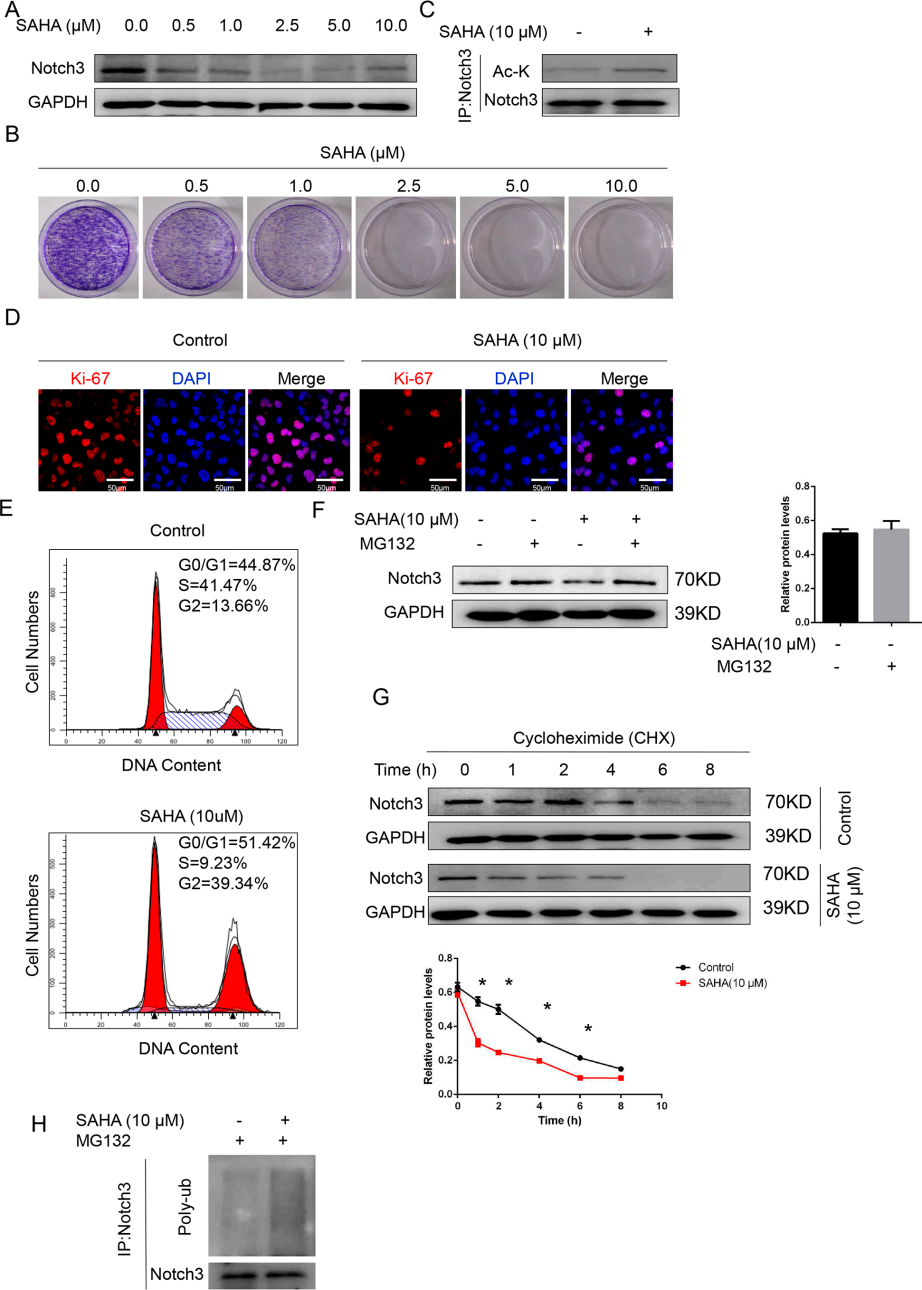
The HDAC inhibitor SAHA decreases Notch3 stability in urothelial cancer cells (**A**) Notch3 protein levels after treatment with SAHA at different concentrations or DMSO (vehicle control), determined by western blot. (**B**) Colony formation efficiency was assessed for T24 cells in presence of SAHA at different concentrations or DMSO (vehicle control). (**C**) T24 cells were treated with 10 μM SAHA or the vehicle alone. Total protein samples were subjected to immunoprecipitation (IP) with anti-Notch3 followed by western blot using anti-acetyl K and anti-Notch3 antibodies. (**D**) Ki-67 levels in T24 cells after treatment with 10 μM SAHA or the vehicle alone, determined by immunofluorescence. Representative photomicrographs are presented from three independent experiments. Scale bar, 50 μm. (**E**) Cell cycle analysis was performed on T24 cells after treatment with 10 μM SAHA or the vehicle alone for 2 days. Then, cells were stained with propidium iodide and analyzed by flow cytometry. (**F**) T24 cells were treated with the proteasome inhibitor MG132 (10 μmol/L). Notch3 protein levels were analyzed by western blot. *P < 0.05 vs. the other groups. (**G**) After T24 cell treatment with cycloheximide (CHX, 100 μmol/L) for the indicated time intervals, Notch3 protein amounts were analyzed by western blot. *P < 0.05 vs. control. (**H**) Ubiquitinated Notch3 was purified from MG132-treated T24 cells. Whole-cell lysates, from T24 cells treated with SAHA (18 h, 10 μM) or the vehicle alone, were immunoprecipitated with anti-Notch3 antibody followed by western blot for ubiquitin detection.

Notch3 acetylation/deacetylation represents a key regulatory switch in the control of Notch signaling, and represents a suitable drug target for Notch3-sustained T-cell acute lymphoblastic leukemia therapy [13]. Accordingly, we hypothesized that *Notch3* expression in urothelial cancer cells might be regulated by HDAC inhibitors. To test this hypothesis, the effect of suberoylanilide hydroxamic acid (SAHA, an HDAC inhibitor) onT24 cells was assessed. Treatment with different doses of SAHA (0.5 to 10 μM) dose-dependently decreased Notch3 protein levels (Figure 6A) and colony formation (Figure 6B). In addition, co-immunoprecipitation demonstrated that SAHA (10 μM) increased the levels of acetylated Notch3 compared to DMSO (vehicle control) (Figure 6C). Interestingly, SAHA (10 μM) decreased the proliferation of T24 cells, as reflected by Ki-67 levels (Figure 6D), and induced cell cycle arrest (Figure 6E).

Protein acetylation is known to prime subsequent ubiquitin-dependent degradation of target proteins [14, 15]. In agreement, addition of the proteasome inhibitor MG132 reversed the reduced Notch3 protein levels observed in SAHA-treated T24 cells (Figure 6F). The half-life of Notch3 in SAHA-treated cells was reduced compared to the vehicle control group (Figure 6G). Therefore, an ubiquitination assay was carried out to assess whether SAHA-induced Notch3 acetylation was followed by ubiquitination-dependent proteasomal degradation. As shown in Figure 6H, treatment with SAHA resulted in markedly increased Notch3 ubiquitination (Figure 6H). Taken together, these findings demonstrated that acetylation regulated NOTCH3 ubiquitination, proteasomal degradation, and function.

## DISCUSSION

Tumor initiation is generally triggered by contextual cues received from the surrounding microenvironment. For example, activation of Notch signaling, which promotes tumor growth, recurrence and drug resistance, will result in poor survival in patients with non-small-cell lung cancer [9], melanomas [16], and breast cancer [17]. However, whether altered *Notch3* expression is associated with outcomes of patients with urothelial cancer remains unknown.

This study demonstrated that Notch3 promotes cancer and is regulated by the level of protein acetylation in urothelial cancer cells. Importantly, high Notch3 levels in urothelial cancer tissues were associated with poor prognosis and short overall survival in patients with urothelial cancer.

To determine whether Notch3 levels affect urothelial cancer progression, the effect of *Notch3* knockdown was assessed in the bladder cancer cell lines T24 and J82. Interestingly, reduced *Notch3* expression resulted in decreased proliferation of T24 and J82 cells *in vitro* and lower tumor progression *in vivo*. These findings indicated that Notch3 is involved in bladder tumorigenesis. To the best of our knowledge, this is the first study identifying Notch3 as a key molecule promoting urothelial cancer tumor progression.

Tumor cells or cancer stem cells acquire resistance to therapeutic agents [18, 19]. In this study, combining *Notch3* knockdown and cisplatin completely abolished drug-resistant colonies of urothelial cancer cells. These findings corroborate the observation that inhibition of Notch activity prevents the development of drug resistance in cancer cells [20]. Thus, Notch signaling may constitute an attractive therapeutic target for the treatment of urothelial cancer.

Accumulating evidence indicates that impairing the acetylation/deacetylation balance in favor of acetylation may be therapeutically exploited in patients with hematological and solid malignancies by using HDAC inhibitors [21–23]. As shown above, the HDAC inhibitor SAHA increased Notch3 protein acetylation levels and decreased *Notch3* expression, in agreement with a previous report [13]. Since SAHA may affect multiple target genes and proteins besides Notch3, it is important to further assess the mechanisms by which SAHA affects Notch3 degradation.

Aoki et al. showed in extrahepatic cholangiocarcinoma that Notch1-3 expression was associated with high tumor grade and poor survival [24]. Hayashi et al. found that Notch2 promotes bladder cancer growth and metastasis through EMT, cell-cycle progression, and maintenance of stemness [25]. Notch signaling is inhibited in urothelial cancer, an effect that is mostly attributed to reduced expression of Notch1 [26]. Nevertheless, Rampias et al. showed that genetic inactivation of Notch signaling leads to Erk1/2 phosphorylation, resulting in tumorigenesis in the urinary tract of mice [27]. Therefore, the role and mechanism of Notch signaling, especially for Notch3, in urothelial cancer remain unclear. As shown above, Notch is involved in urothelial cancer progression via an acetylation mechanism, but more studies are needed to identify the exact mechanisms. The carcinogenic mechanism of Notch signaling could be associated with multiple pathways. Giovannini et al. found in liver cancer that decreased Notch3 expression could increase the p53 levels, inducing DNA damage, increasing adriamycin-induced apoptosis, and blocking cell cycle progression in liver cancer cells; these findings suggested associations of high Notch3 expression and tumor metastasis, vascular invasion, and satellite focus [28]. Inhibiting Notch3 promotes GSK313 phosphorylation and downregulates p21, increasing the toxic effects of sorafenib on liver cancer cells [29]. Notch3 signaling is activated by the canonical Wnt/β-catenin pathway and results in altered proliferation and apoptosis of tumor cells [30]. Meunier et al. [31] showed that Notch3 is involved in the response to hypoxia of prostate cancer cells and that HES-1 was not involved in this association. Aoki et al. [24] showed a tendency of HES-1 to be associated with survival of patients with extrahepatic cholangiocarcinoma. These findings indicated that Notch3 signaling is involved in most tumors, with an important role in tumor development, progression, and prognosis. However, Cui et al. found that *Notch3* expression in normal tissues is higher than in tumor cells [32]. Notch3 signaling is not an isolated pathway; actually, it is affected by other factors and signaling pathways. Therefore, more studies are needed to provide a clear role for this pathway in urothelial cancer.

Taken together, *Notch3* overexpression enhances growth and chemoresistance in urothelial carcinoma, indicating that Notch3 protein levels may serve as a prognostic marker in human urothelial cancer.

## MATERIALS AND METHODS

### Tissue samples and cell lines

Fresh tumor specimens and non-tumor bladder tissue samples were obtained from 59 patients who had undergone surgical resection for primary urothelial cancer between February 2007 and November 2012 at the Department of Urology, Southwest Hospital, Third Military Medical University (Chongqing, China). All tissue samples were pathologically confirmed to be primary urothelial carcinoma; surrounding normal tissues were included as well. Written informed consent was obtained from each patient; the study was approved by the Institutional Review Board of the Third Military Medical University.

The patients included 50 men and 9 women with an average age of 68 years (range: 44–85 years). None of them had had radiotherapy or chemotherapy; those with smoking history of > 20 cigarettes a day (considered as heavy smokers), or a history of alcohol abuse or contact with aromatic organic compounds were excluded. The criteria for tumor classification were those of the UICC-TNM. Histological grading was based on the 2004 World Health organization/International Society of Urological Pathology classification [33]. The baseline clinical and pathological characteristics of the patients are summarized Table 1.

The human bladder cancer cell lines T24 and J82 were purchased from Shanghai Cell Collection (Shanghai, China) and cultured in RPMI 1640 (Gibco, Paisley, Scotland, UK) with 10% FBS (Gibco) at 37°C in a humidified atmosphere containing 5% CO_2_.

### Lentivirus production and cell line transduction

Lentiviruses were prepared in 293T packaging cells via transfection with a four-plasmid system. Transfections were performed in 100-mm diameter plates. Packaging cells were seeded at 3.5 × 10^5^ cells per plate in DMEM containing 10% FBS 24 h before transfection and grown at 37°C with 5% CO_2_. DNA for transfection was prepared by mixing 15 μg of shRNA or gene-encoded plasmid, 4 μg pRRE, 3 μg pREV, and 6 μg pMD2. These reagents were mixed with 50 μL CaCl_2_ (1.25 M) and 500 μL of 2× HBS in a final volume of 1 mL. Lentiviral supernatants from 48 and 72 h were pooled and stored at –80°C.

For shRNA or gene-encoded lentivirus-mediated knockdown experiments, cells were infected with the same viral MOI. After an overnight incubation, the medium was refreshed and hexadimethrine bromide was added 72 h post-infection at 2 μg/mL. Protein expression was assessed by immunoblotting after 72 h of culture in the selection medium.

The following sequences were used for shNotch3 and control cells: Scrambled shRNA, 5′-CGT ACG CGG AAT ACT TCG A-3′; *Notch3* shRNA1, 5′-GCA TGA AGA ACA TGG CCA A-3′; *Notch3* shRNA2, 5′-ATG CCT AGA CCT GGT GGA CAA-3′.

### Cell counting kit-8 (CCK-8) assay

The viability of shNotch3 cells was quantified using Cell Counting Kit-8 (26992; Sigma-Aldrich, St. Louis, MO, USA), according to the manufacturer's instructions. Briefly, 5000 cells were seeded in each well of a 96-well plate. After treatment with Notch3 shRNA, 10 μL of CCK-8 solution was added per well and incubated for 4 h. Absorbance at 450 nm was measured on a microplate reader.

### Cell cycle analysis

T24 cells treated with 10 μM SAHA (Vorinostat; MedChem Express Co., Monmouth Junction, NJ, USA) or vehicle (DMSO) for 2 days were stained with propidium iodide and subjected to flow cytometry following standard protocols. Briefly, cells were fixed with 10 mL of 70% ethanol at –20°C overnight, stained with 0.5 mL of propidium iodide/RNase staining buffer for 15 min at room temperature, and analyzed by flow cytometry.

### Immunohistochemistry

Tissue specimens were fixed with formalin and embedded in paraffin. A tissue array block containing both urothelial cancer and non-tumor tissue samples was constructed. Immunohistochemistry was performed as described elsewhere [34]. The expression levels of Notch3 were scored semi-quantitatively based on staining intensity (SI) and percentage of positive cells (PP) using the immunoreactive scores (IRS)= SI × PP. SI was defined as: 0 = negative; 1 = weak; 2 = moderate; and 3 = strong. PP was defined as 0 = 0%; 1 = 0–25%; 2 = 25–50%; 3 = 50–75%; and 4= 75–100%. Cases were divided into two groups based on IRS of Notch3 staining, as proposed previously [35]: IRS ≥ 4, high expression group; IRS < 4, low expression group.

### Immunofluorescence

T24 cells were cultured on glass cover-slips in 24-well plates, fixed with 4% paraformaldehyde, and permeabilized with 0.1% Triton X-100 in PBS for 5 min at room temperature. Cells were then blocked with 10% FBS (Gibco) in PBS for 30 min at room temperature. Cover-slips were incubated with a primary antibody against Ki-67 (Maixin-Bio, Fuzhou, China). Donkey anti-rabbit IgG Alexa Fluor 647 (Molecular Probes, Waltham, MA USA) was used as the secondary antibody. Cells were further washed in PBS and mounted with Vectashield mounting medium (Vector Laboratories, Burlingame, CA, USA) containing DAPI for counterstaining. Cells were analyzed by fluorescence microscopy.

### Tumor formation assay

NOD/SCID mice (male, 3–5 weeks old) were maintained under pathogen-free conditions at the animal facility of the Third Military Medical University. Mouse experiments were approved by the Institutional Animal Care and Use Committee at the Third Military Medical University; 2 × 10^6^ T24 and J82 cells, respectively, treated with Notch3-shRNA or scrambled shRNA, were resuspended in serum-free medium, and subcutaneously injected into the right flank of NOD/SCID mice. Four weeks after cell injection, mice were euthanized, and tumor volumes and weights were measured. The xenograft tumors were fixed with 4% paraformaldehyde (Sigma-Aldrich), embedded in paraffin, and sectioned at 4–5 μm. The xenograft tumors were observed by microscopy. Protein expression was detected by EnVision immunohistochemistry (Dako, Glostrup, Denmark).

### Colony formation assay

Briefly, 1 × 10^4^ T24 or J82 cells were seeded in 10-cm tissue culture plates, and treated with SAHA at different concentrations or DMSO, in 1640 medium supplemented with 10% of FBS for 15 days. The colonies were fixed with 4% formaldehyde and stained with 0.1% crystal violet (Sigma-Aldrich). To assess drug sensitivity, cells were exposed to cisplatin (5 μM) for 15 days, fixed, and stained with 1% crystal violet. Colonies were counted.

### Western blot

Cells were treated with SAHA (24 h), MG132 (Abcam, Cambridge, UK; 6 h), and cycloheximide (Sigma-Aldrich; indicated time intervals), respectively. Western blot was performed as previously described [18, 34]. Briefly, cells were harvested and lysed for 30 min at 4°C in RIPA lysis buffer (Beyotime, Shanghai, China). Total protein samples (equal amounts) were separated by 10% SDS-polyacrylamide gel electrophoresis (PAGE) and transferred onto PVDF membranes (Millipore, Billerica, MA, USA). After blocking in 5% milk for 2 h at room temperature, the membranes were incubated with the primary antibody (rabbit anti-Notch3 antibody; Cell Signaling Technology, Danvers, MA, USA; catalog No 2889) overnight at 4°C. Then, anti-rabbit IgG horseradish peroxidase-conjugated secondary antibody was added at 1:2000, and immuno-complexes were visualized using the Super Signal West Femto Chemiluminescent Substrate (Pierce, Rockford, IL, USA). For quantification, signals were normalized to GAPDH (Cell Signaling Technology) with the Gene Tools image analysis program (Syngene, Cambridge, UK).

### Co-immunoprecipitation

Cells were lysed in RIPA buffer (Beyotime) for 15 min on ice. The resulting lysates were centrifuged at 14,000 rpm at 4°C for 15 min. The supernatants containing the relevant proteins were incubated with Notch3 primary antibody (1:200 dilution; Cell Signaling Technology) at room temperature for 2 h. To detect antibody complexes, 100 μL of protein A + G agarose (Beyotime) were incubated with the supernatants at 4°C overnight. Agarose-antibody complexes were harvested by centrifugation at 14,000 rpm for 5 s, washed thrice with PBS, and recovered in 1× SDS-PAGE buffer in a boiling water bath for 5 min. The immunoprecipitates were analyzed by western blotting with antibodies against Ac-K (1:400 dilution; Millipore) and ubiquitin (1:400 dilution; Millipore).

### Statistical analysis

The Student's *t* test was used to compare experimental groups. Kaplan-Meier survival curves and log-rank test were used to estimate patient survival and differences between groups. SPSS 10.0 and GraphPad Prism 5.0 were used for statistical analyses. *P* < 0.05 was considered statistically significant.
